# Amino Acid-Dependent Alterations in Cell Wall and Cell Morphology of *Deinococcus indicus* DR1

**DOI:** 10.3389/fmicb.2019.01449

**Published:** 2019-07-03

**Authors:** Deepika Chauhan, Pulkit Anupam Srivastava, Barbara Ritzl, Ragothaman M. Yennamalli, Felipe Cava, Richa Priyadarshini

**Affiliations:** ^1^Department of Life Sciences, School of Natural Sciences, Shiv Nadar University, Greater Noida, India; ^2^Department of Biotechnology and Bioinformatics, Jaypee University of Information Technology, Waknaghat, India; ^3^Laboratory for Molecular Infection Medicine Sweden, Department of Molecular Biology, Umeå Centre for Microbial Research, Umeå University, Umeå, Sweden

**Keywords:** *Deinococcus indicus*, morphological alterations, amino acids, cell wall, muropeptides

## Abstract

*Deinococcus radiodurans* exhibits growth medium-dependent morphological variation in cell shape, but there is no evidence whether this phenomenon is observed in other members of the Deinococcaceae family. In this study, we isolated a red-pigmented, aerobic, *Deinococcus indicus* strain DR1 from Dadri wetland, India. This *D. indicus* strain exhibited cell–morphology transition from rod-shaped cells to multi-cell chains in a growth-medium-dependent fashion. In response to addition of 1% casamino acids in the minimal growth medium, rod-shaped cells formed multi-cell chains. Addition of all 20 amino acids to the minimal medium was able to recapitulate the phenotype. Specifically, a combination of L-methionine, L-lysine, L-aspartate, and L-threonine caused morphological alterations. The transition from rod shape to multi-cell chains is due to delay in daughter cell separation after cell division. Minimal medium supplemented with L-ornithine alone was able to cause cell morphology changes. Furthermore, a comparative UPLC analysis of PG fragments isolated from *D. indicus* cells propagated in different growth media revealed alterations in the PG composition. An increase in the overall cross-linkage of PG was observed in muropeptides from nutrient-rich TSB and NB media versus PYE medium. Overall our study highlights that environmental conditions influence PG composition and cell morphology in *D. indicus*.

## Introduction

Deinococcaceae family is among the few groups of bacteria that are ubiquitously distributed in the environment. The members of this family are aerobic, non-spore forming, chemo-organotrophs that display extreme resistance to UV/gamma radiation as well as desiccation (Cox and Battista, 2005). *Deinococcus* sp. have been isolated from distinct locations varying from extreme to common habitats such as Antarctic soil, deserts, hot springs, air, radiation sites, heavy-metal contaminated soil, sewage, plant rhizosphere, and human stomach (Ito et al., 1983; Ferreira et al., 1997; Ellis et al., 2003; Hirsch et al., 2004; De Groot et al., 2005; Bik et al., 2006; Lai et al., 2006; Weon et al., 2007; Asker et al., 2008).

Although most of the members of *Deinococcus* genus are Gram-positive coccoids, a few members, including *D. indicus*, are Gram-negative rods. Examples include *D. deserti* (Suresh et al., 2004; De Groot et al., 2005); *D. ficus* (Lai et al., 2006); *D. grandis* (Oyaizu et al., 1987); *D. indicus* (Suresh et al., 2004); *D. aquaticus* and *D. caeni* (Im et al., 2008); and *D. depolymerans* (Asker et al., 2011). Members of family Deinococcaceae have a complex cell envelope. *D. radiodurans* has an unusual multilayered cell envelope, which includes a thick peptidoglycan layer, an outer membrane like lipid layer, and a S-layer (Battista, 1997; White et al., 1999). Bacterial outer membrane proteins require either the beta-barrel assembly machinery (BAM) for their correct folding or the translocation and assembly module (TAM). The TAM consists of two components TamA and TamB that form a complex essential for assembly of several outer membrane proteins (Webb et al., 2012). Recent study identified a TamB homolog in *D. radiodurans* that plays a role in maintaining cell envelope integrity (Yu et al., 2017). Genome mining revealed that *D. indicus* also harbors a TamB homolog, suggesting that *D. indicus* may display similar cell envelop properties. Some members of Deinococcaceae family also exhibit morphological transition and are found to exist in a number of morphotypes in response to environmental conditions (Wainwright, 1997). Multiple cell formation was previously reported in *Deinococcus radiodurans* where it was seen that cell growth and division took place without separation, creating a cluster of cells (Chou and Tan, 1991).

In this study, we report the characterization of a red-pigmented, Gram-negative rod-shaped bacterium previously isolated from the wetland of Dadri, India (Chauhan et al., 2017). The bacterium was identified as belonging to the genus *Deinococcaceae* and was designated as *Deinococcus indicus DR*1 (referred as *D. indicus* in the manuscript). Our studies show nutrition-induced morphotypes in *D. indicus* which manifests as growth in multi-cell chains with increased cell size in nutrient-rich media in contrast to its existence as short rods in minimal or nutrient-depleted media. A role for casamino acids (CAA) in the growth medium is indicated for the observed phenotype. Addition of amino acid mixture to the minimal medium was able to recapitulate the multi-cell chaining phenotype, suggesting that concentration of amino acids in the growth medium induced morphological alterations. Moreover, addition of only L-ornithine to minimal medium was sufficient to cause cell morphology changes. Furthermore, a comparative UPLC analysis of PG fragments isolated from *D. indicus* cells propagated in different growth media revealed alterations in PG composition. The overall cross-linkage of PG was increased in muropeptides extracted from nutrient-rich TSB and NB media compared to PYE medium. Our study showed an unexpected plasticity in cell wall of *D. indicus*.

## Materials and Methods

### Sample Collection

Water samples were collected in spring (April 2014) from the wetland of Dadri located in the north western region of Uttar Pradesh, India (coordinates 28°31′30.7″N 77°34′40.1″E). The average pH of water samples collected was ∼7.6 and the average temperature maintained in the water body throughout spring season was recorded as 30°C. Samples were collected in sterile glass bottles and immediately taken for further processing. Two liters of the collected water sample was filtered in a batch of 100 ml through a 0.22-μm-pore size filter (Millipore Corp.). Filter papers having the concentrated samples were kept over different media plates (PYE, R2A, LB, M2G, and M9) in triplicates and peeled off after 2 h. Plates were incubated overnight at 30°C. The next day, pinkish-red colonies were selected and streaked on fresh plates.

### Media and Growth Conditions

Cells were grown under agitation (200 rpm) at 30°C in various growth media. Seven different media were used to perform experiments: PYE medium (Poindexter, 1964) (2.0 g L^-1^ peptone, 1.0 g L^-1^ yeast extract, 1 ml L^-1^ 1 M MgSO_4_, and 1 ml L^-1^ 1 M CaCl); Luria Bertani broth (LB) (Bertani, 1951) (10.0 g L^-1^ casein enzymic hydrolysate, 5 g L^-1^ yeast extract, and 10 g L^-1^ NaCl); minimal salts medium, M63 (Pardee et al., 1959) supplemented with 1 mM MgSO_4_, 0.2% glucose, and 0.5% CAA; defined minimal M2G medium [0.87 g L^-1^ Na_2_HPO_4_, 0.54 g L^-1^ KH_2_PO_4_, 0.50 g L^-1^ NH_4_Cl, 0.2% (wt/vol) glucose, 0.5 mM MgSO_4_, 0.5 mM CaCl_2_, and 0.01 mM FeSO_4_]; Nutrient broth (NB) (Difco) (15.0 g L^-1^ peptone, 3.0 g L^-1^ yeast extract, 6.0 g L^-1^ NaCl, and 1.0 g L^-1^ glucose); and Tryptone soy broth (TSB) (Difco) (17.0 g L^-1^ pancreatic digest of casein, 3.0 g L^-1^ papaic digest of soyabean meal, 5.0 g L^-1^ NaCl, 2.5 g L^-1^ K_2_HPO_4_, and 2.5 g L^-1^ dextrose). All media were purchased from Hi-Media Laboratories (Mumbai, India) and Difco.

### DNA Isolation and 16S rRNA Gene Sequencing

Genomic DNA was isolated as described previously (Udupa et al., 1994). Briefly, pellet of 10 ml culture was resuspended in 450 μl TE buffer after 15 min treatment in 95% ethanol. Cells were then treated with 0.1 mg lysozyme (Sigma–Aldrich, St. Louis, MO, United States) followed by incubation for 30 min at 30°C. In addition, cells were treated with 25 μl 10% SDS and 2.5 μl proteinase K (20 mg/ml; Sigma–Aldrich, St. Louis, MO, United States) for 12 h at 56°C. DNA was extracted by phenol–chloroform treatment and stored in TE buffer (pH 8.0) at 4°C.

For initial identification, isolated DNA was amplified by PCR with the universal primers targeting 16S rRNA gene sequence of bacteria – 27F: 5′-AGAGTTTGATCMTGGCTCAG-3′ and 1492R: 5′-TACGGYTACCTTGTTACGACTT-3′. In addition, *D. indicus*-specific primers were designed using its partial 16S rRNA sequence for further verification (FP: 5′-AGGGTTTGATCCTGGCTC-3′; RP: 5′-GGGCGGTGTGTACAAGGC-3′). The following cycling conditions were used for PCR: 94°C for 10 min (94°C for 40 s, 56°C for 1 min, 74°C for 2 min) × 30 cycles, 74°C for 10 min (Eppendorf Thermal Cycler). Amplicons from PCR reaction were purified using PCR purification kit (Qiagen) and sent for sequencing (SciGenom Labs Private Ltd., Kerala, India). The sequence similarity was analyzed with BLAST tool on NCBI^1^ which showed 99% similarity with *D. indicus*. Further, by multiple sequence alignment (Clustal Omega)^2^, the number of mismatches among top hits was compared with partial 16S rRNA sequence of *D. indicus* Wt/1a strain.

### Growth and Cell Morphology Assay

*Deinococcus indicus* cells were grown overnight in PYE medium and then re-inoculated in 5 ml fresh media (PYE, LB, M2G, M63, NB, and TSB), respectively. Cells were harvested at optical density (OD_600_) of 0.6, and imaged by DIC microscopy. Cell length was measured at 0.6 OD_600_ (*n* = 200) by Nikon Eclipse Ti analysis tool and plotted using GraphPad Prism software. To check the effect of media components on morphological variation in *D. indicus*, cells were grown with varying concentration of CAA. Overnight grown cells, previously inoculated in M2G medium at 30°C, were re-inoculated in fresh M2G with increasing concentration of CAA (0.1, 0.5, and 1%). Cell morphology was analyzed at the log phase using Nikon Eclipse Ti-E inverted microscope. All experiments were performed in triplicate.

### Amino Acid Assay

*Deinococcus indicus* cells were grown overnight in M2G medium at 30°C. Using this overnight culture, cells were re-inoculated in M2G medium containing specific combination of amino acids as described in Supplementary Tables S1, S2. Control samples were grown in M2G medium without amino acids. After 24 h of incubation, cells were imaged in DIC using 1% agarose slides made in M2G medium. On the basis of morphology, cells were counted and plotted accordingly. Cell morphology was also analyzed with various combinations of amino acids using standard concentrations and 11 different amino acid combinations were examined for the diagnosis of auxotrophs according to the Manual for Advanced Molecular Genetics (Davis et al., 1980). The combination of all 20 amino acids was maintained at 0.1% concentration whereas L-ornithine concentration in the medium was kept equivalent to L-lysine concentration (0.005%). Cells were imaged in DIC with 100× oil-immersion objective.

### Microscopy and Time-Lapse Imaging

*Deinococcus indicus* was inoculated in PYE medium and incubated overnight at 30°C. Next day, culture was re-inoculated in NB medium (1:100 dilution) and grown to an optical density of 0.1. Cells (5 μl) were placed over 1% agarose-coated slides made in NB medium and incubated at room temperature for 10 min to immobilize the cells. Time-lapse was performed for 8 h using a 100× oil-immersion objective, in DIC on a Nikon Eclipse Ti-E inverted microscope. Heating platform was used to maintain temperature at 30°C.

### DAPI and FM 4-64 Staining

Cells were grown overnight in PYE, LB, NB, or TSB media at 30°C, re-inoculated next morning to mid-log phase and 1 ml of culture was centrifuged for 3 min at 2000 × *g*. Pellets were washed with 1× phosphate buffer saline (PBS), resuspended, and fixed in 70% ethanol for 2 min at room temperature. Fixed cells were spun and washed again with 1× PBS. Finally, FM 4-64 (4 μg/ml) was added and the cells were incubated for 15 min in dark, then DAPI (1 mg/ml) was added to the cells (1:1000 dilution) and left for additional 15 min at room temperature in dark. After final wash with 1× PBS cells were observed by microscopy.

### Image Analysis

#### Cell Length and Width Measurement After Amino Acid Treatment

Using Fiji (ImageJ) software (Schindelin et al., 2012), region of interest (ROI) was drawn using the line tool over DIC images of 50 cells from different amino acids treated sets. Cells were selected from two separate experiments. From these ROI, average length and width of 50 individual cells were obtained. Next, the standard deviation was calculated for each set and represented accordingly.

#### Line Scan for Fluorescence Intensity Analysis

Using Fiji (ImageJ) software (Schindelin et al., 2012), DAPI- and FM 4-64-stained cells were scanned for fluorescence intensity. Individual cells from each growth media (PYE, LB, NB, and TSB) were scanned using segmented line tool. The selected line scan was restored for other channel for unbiased fluorescence intensity measurement. Scan values were plotted individually for cells grown in separate media comparing different channels.

### Scanning Electron Microscopy (SEM)

Cells were grown to mid-log phase in PYE, LB, NB, and TSB media, respectively, harvested, and resuspended in 1× PBS. The cells were then fixed primarily with 2.5% glutaraldehyde and postfixed in 1% OsO_4_. Further, cells were prepared as previously described (Morris, 1965). Cell imaging was performed using a Carl Zeiss EVO 40 scanning electron microscope.

### Peptidoglycan Isolation and Analysis

For the peptidoglycan profile and analysis, *D. indicus* DR1 strain was grown in three different media and harvested. Peptidoglycan purification was carried out as previously described in Desmarais et al. (2013) and Alvarez et al. (2016) with some minor changes. Briefly, the cell pellets of *D. indicus* were boiled in an equal volume of 5% (w/v) SDS for an hour and stirred overnight at 37°C. The sacculi were washed repeatedly with MilliQ water by ultracentrifugation (150,000 × *g*, 13 min, 20°C Optima^TM^ Max Ultracentrifuge, Beckman Coulter, CA, United States). The clean sacculi were digested with muramidase (Cellosyl 100 μg/ml) overnight at 37°C. The muramidase digestion was stopped by heat-inactivation at 100°C. Coagulated proteins were removed by centrifugation (20,000 × *g* for 15 min). To reduce the samples, the supernatants were adjusted to pH 8.5–9.0 with borate buffer, followed by the addition of freshly prepared NaBH_4_ solution to a final concentration of 10 mg/ml. After 30 min incubation at room temperature, samples were adjusted to pH 3.5 with phosphoric acid. Muropeptides were separated by UPLC on a Waters UPLC system (Waters, Milford, MA, United States) equipped with a Kinetex C18 UPLC Column, 130 Å, 1.7 μm, 2.1 mm × 150 mm (Waters, Milford, MA, United States) and a dual wavelength absorbance detector. Elution of muropeptides was detected at 204 nm. Muropeptides were separated at 45°C using a linear gradient from Buffer A [formic acid 0.1% (v/v)] to Buffer B [formic acid 0.1% (v/v), acetonitrile 40% (v/v)] in an 18 min run with 0.250 ml/min flow.

To identify the muropeptides as well as their chemical structure, samples were run by UPLC–MS (UPLC system interfaced with a Xevo G2/XS Q-TOF mass spectrometer from Waters, Milford, MA, United States) using an organic separation method. Visualization and acquisition of the data was performed by using UNIFI software platform (Waters, Milford, MA, United States).

## Results

### Growth Medium-Dependent Morphological Changes in *D. indicus*

We previously isolated a red pigmented, aerobic, Gram-negative, rod-shaped bacterium from the water samples of Dadri wetland, situated in Uttar Pradesh, India (Chauhan et al., 2017). Sequence alignment revealed 100% identity with *D. indicus* strain wt/1a 16S ribosomal sequence. The whole genome of this bacterium was sequenced and the bacterium was named *D. indicus* strain DR1 (Chauhan et al., 2017) and is referred as *D. indicus* in the rest of the manuscript. The family Deinococcaceae consists of non-spore forming bacteria which survive in ionizing radiations. *D. indicus* was first isolated from an arsenic contaminated aquifer in West Bengal, India (Suresh et al., 2004).

**FIGURE 1:**
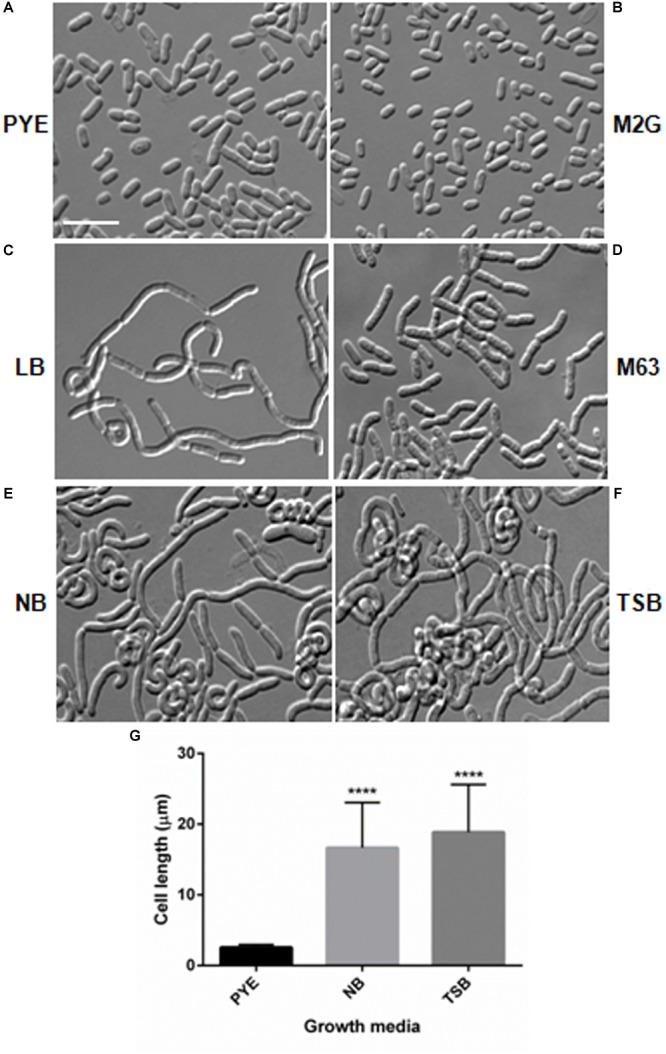
Growth medium dependent morphological changes in *D. indicus*. DIC micrographs of *D. indicus* cells grown in varied growth media showed different morphology. For microscopy, cells were grown in **(A)** PYE, **(B)** M2G, **(C)** LB, **(D)** M63, **(E)** NB, and **(F)** TSB growth medium at 30°C for 18 h. **(G)** Cell length was measured (*n* = 205) and compared with PYE incubated cells indicating a significant increase in the cell length in NB and TSB growth media (*p* < 0.001). Scale bar, 10 μm.

During growth studies we observed that *D. indicus* exhibited different cell morphologies. Further examination revealed that *D. indicus* displayed small rod-shaped cells in PYE growth medium and multi-cell chains in LB, TSB, and NB media (Figure 1). We then grew *D. indicus* cells in different growth media and observed cell morphology. In both PYE and M2G media cells were short rods (Figure 1A,B) and in LB medium cells were elongated and attached as chains (Figure 1C). In M63, NB, and TSB media multi-cell chains were observed, which were further coiled on each other (Figure 1D–F). Our results suggested that *D. indicus* displayed different morphotypes in different growth media. It is plausible that cell morphology changes could be a result of differential growth rate of *D. indicus* in various growth media. Generation time of *D. indicus* in various media was measured and it was found to be approximately 120 min in PYE and TSB, 150 min in LB, and 90 min in NB (Supplementary Table S4). As both in PYE and TSB the generation time was 120 min, and cells are rod shaped in PYE and multi-cell chains in TSB; it was concluded that growth rate was not the cause of the phenotype observed.

### Growth in Nutrient-Rich Media Leads to Inhibition of Cell Separation

**FIGURE 2:**
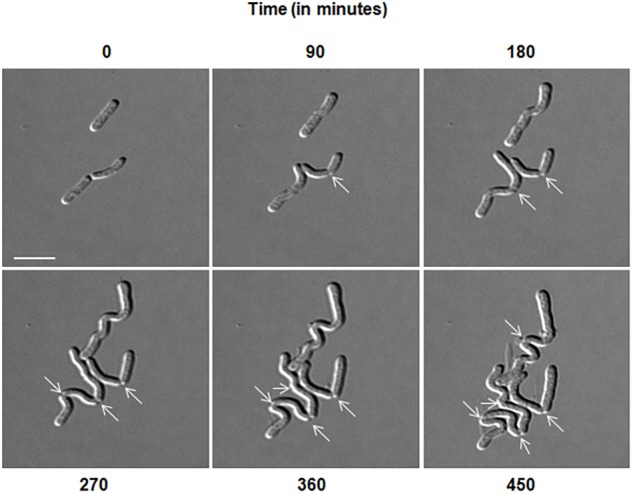
Delayed cell separation of daughter cells observed in *D. indicus.* Time-lapse was performed in DIC for 8 h in nutrient broth medium. After 90 min, rod shaped cells started elongating and formed irregular chains. Arrows indicate formation of new division sites and separation inability. Scale bar, 5 μm.

**FIGURE 3:**
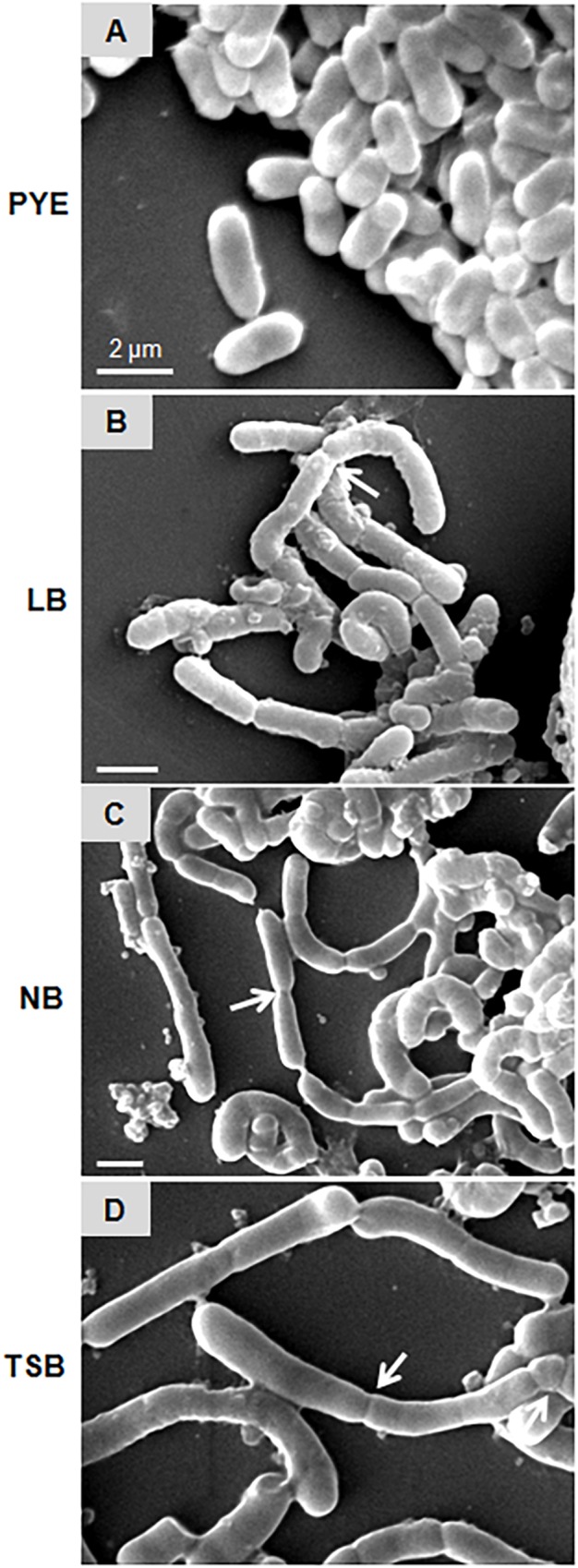
Scanning electron micrographs represent *D. indicus* cell attachment pattern in different growth media. *D. indicus* display rod morphology in PYE medium **(A)** whereas in LB **(B)**, NB **(C)**, and TSB growth media **(D)** multicellular chains were observed. Arrows indicate sites of cell attachment. Scale bar, 2 μm.

**FIGURE 4:**
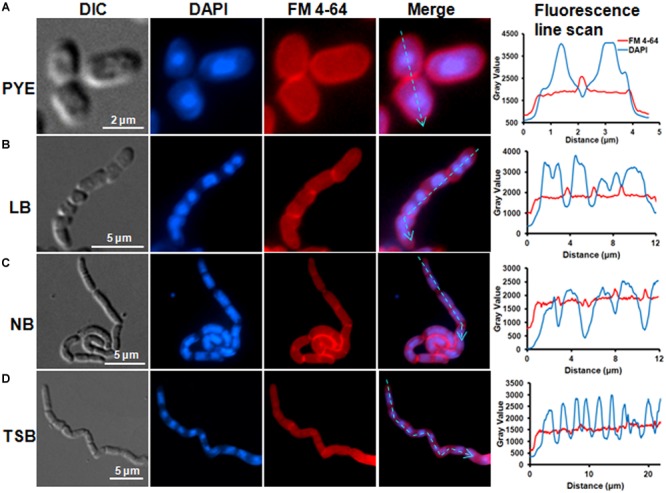
FM4-64 and DAPI staining showed daughter cell attachment at division sites. Cells were grown in **(A)** PYE, **(B)** LB, **(C)** NB, and **(D)** TSB growth media at 30°C to an OD_600_ of 0.4–0.5 followed by inner membrane and nucleic acid labeling by FM4-64 and DAPI. Line scan analysis of cells grown in various media showed the distribution of DAPI and FM 4-64 along the cell length. Fluorescence intensities are represented as plots next to each panel. The cyan arrowed lines correspond to sections that were scanned to determine the fluorescence intensity.

Microscopic analysis of *D. indicus* cells grown in LB, TSB, or NB media showed cells in chains, suggesting that some stage of cell division was inhibited. To get a closer look, we observed growth of *D. indicus* cell on TSB medium by time-lapse microcopy. In the initial stages the cells grew and divided normally and after 90 min, the cells started to elongate (Figure 2). The elongated cells were connected to each other at the old division sites (Figure 2 and Supplementary Video S1). Even after 4 h daughter cells were not separated, suggesting a delay in cell separation. To confirm that the daughter cells remain attached at the division sites in multi-cell chains, we examined *D. indicus* cells grown in various growth media by SEM. Cells in PYE medium were rod shaped (Figure 3A), while cells grown in LB, NB, and TSB media were in chains (Figure 3B–D). Cells in chains were connected at the division site, showing clear indentations (Figure 3B–D), suggesting that cell division is occurring but cell separation is not complete. To probe further which stage of cell division is inhibited in *D. indicus* cells, we grew cells in different growth media and stained them with DAPI and FM 4-64, a lipophilic membrane dye. FM 4-64 dye has been used previously to stain membrane of *D. radiodurans*, and FM 4-64 dye labeling was also observed at the division sites (Nguyen et al., 2009). As evident from Figure 4A, in PYE single rod-shaped cells and dividing cells with FM 4-64 staining at the midcell were observed. In LB, NB, and TSB media, strings of cells were attached together and in some cases FM 4-64 staining was retained between two cells at the old-division site (Figure 4B–D). Fluorescence intensity profiles of dividing cell obtained from PYE medium reinforced these observations (Figure 4A). Cell division sites in these cells were identified by the coincident combination of a high intensity red peak (FM 4-64) coupled with a trough of low intensity between blue (DAPI) peaks (Figure 4A). In multi-cell chains formed in NB, TSB, and LB media, multiple high intensity blue peaks (DAPI signaling for the nucleoid) were observed along with multiple division sites (high intensity red peak coupled with a trough of low intensity between blue peaks) distributed in a repeating pattern along the lengths of the filaments (Figure 4B–D). Thus, our results suggest that in nutrient-rich media decoupling of cell division and cell separation leads to the formation of multi-cell chains.

**FIGURE 5:**
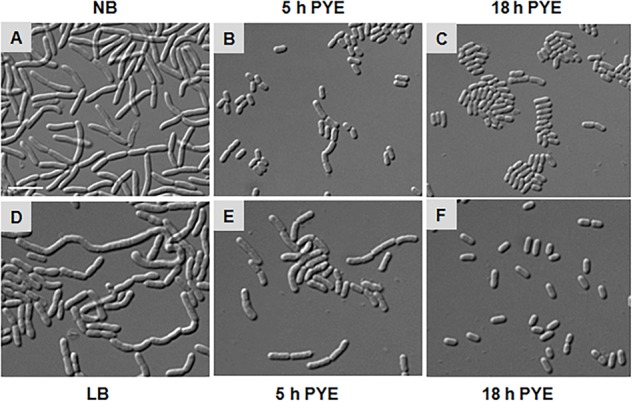
Morphological alterations observed in *D. indicus* during transition from nutrient-rich to nutrient-depleted growth media. Overnight culture of *D. indicus* in NB **(A)** and LB **(D)** media was washed twice and re-inoculated in PYE medium and observed under microscope after 5 **(B,E)** and 18 h **(C,F)** incubation at 30°C. Scale bar, 10 μm.

As growth of *D. indicus* cells in nutrient-rich growth media delayed cell separation, it is plausible that when these multi-cell chains are transferred back to nutritionally poor medium, they would resume growth as rod-shaped cells. To test, we grew cells overnight in NB and LB media and then transferred them into PYE medium (Figure 5A,D). After 5 h of growth in PYE, majority of the cells were rod shaped and only a few short multi-cell chains were observed (Figure 5B,E). After overnight growth in PYE medium all the cells were rod shaped (Figure 5C,F), indicating that there was no lag in cell separation. We also observed this phenomenon by time-lapse microscopy, and when cells grown in NB media were placed on PYE pad they grew as rod-shaped cells (Supplementary Video S2). In this case there was no lag in daughter cell separation after cell division, suggesting that the morphology of *D. indicus* is influenced by the growth medium.

### Concentration of Amino Acids in the Medium Influenced Morphological Changes in *D. indicus*

**FIGURE 6:**
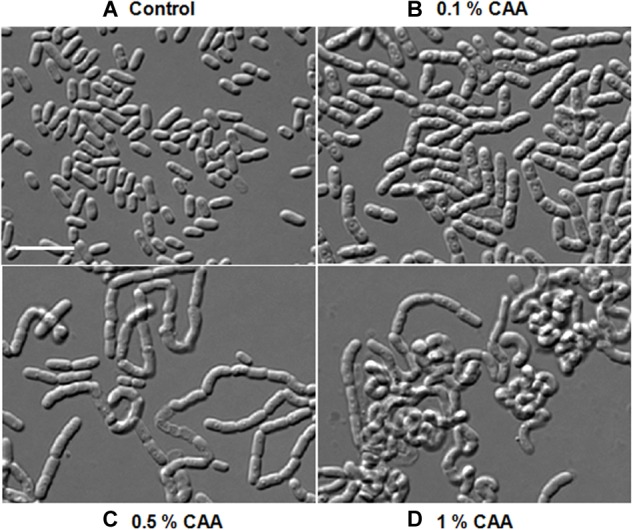
*D. indicus* formed multi-cell chains in the presence of casamino acid (CAA) in growth media. Cells were grown at 30°C for 18 h in M2G medium with increasing concentration of CAA. **(A)** M2G medium without CAA (control), **(B)** M2G with 0.1% CAA, **(C)** M2G with 0.5% CAA, and **(D)** M2G with 1% CAA. Cells started elongating and formed convoluted multi-cell chains on successive addition of CAA. Scale bar, 10 μm.

As morphological alterations were dependent upon some changes in the growth media composition, we analyzed the component of various media. PYE, LB, TSB, and NB are complex growth media containing, peptone, beef extract, or tryptone. M2G and M63 are minimal media, whereas M63 contains CAA, which are lacking in the former. Cells were rod shaped in M2G and multi-cell chains in M63, suggesting that the concentration of CAA in the growth media may be influencing the morphological alterations. To confirm this we added increasing amount of CAA in M2G medium and grew *D. indicus* cells (Figure 6). *D. indicus* cells started to chain upon addition of 0.1% CAA and these chains began to coil as the concentration was increased to 1% (Figure 6A–D). M2G containing more than 1% CAA severely affected the growth of *D. indicus* cells. Taken together our data suggest that, in the presence of CAA, *D. indicus* grows and divides but delays cell separation, resulting in the formation of multicellular chains. LB and TSB contain NaCl, while PYE medium is devoid of NaCl. It is possible that the change in cell shape is influenced by salt concentration in the growth medium. To investigate this further, we grew *D. indicus* cells in LB and TSB media devoid of NaCl and cells were still attached together in chains suggesting that the phenotype observed was not due to the presence of sodium chloride in the growth medium (Supplementary Figure S1). Moreover, addition of NaCl in the PYE medium did not alter cell shape (Supplementary Figures S1A,B).

**FIGURE 7:**
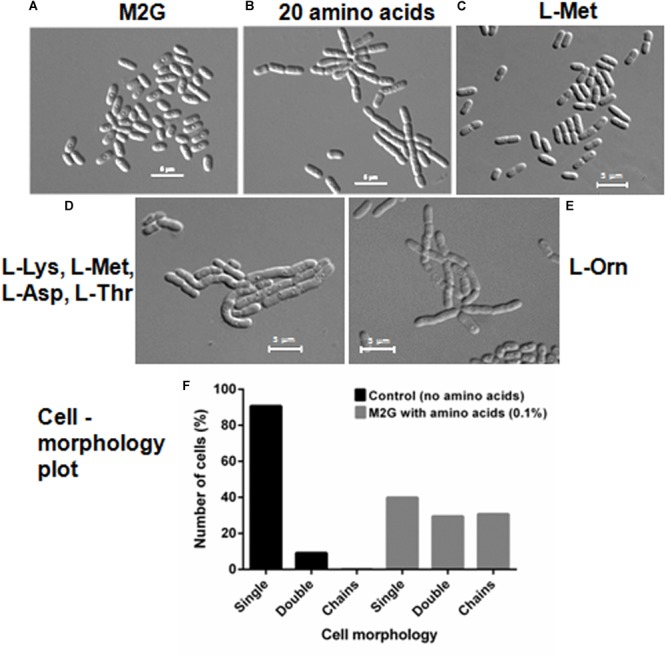
Addition of amino acids in M2G medium recapitulates cell chaining phenotype. Cells were re-inoculated in M2G medium from overnight grown culture without amino acid **(A)** and with combination of individual amino acids (0.1 %) **(B)**, followed by incubation at 30°C for 24 h. Number of cells were counted and plotted on the basis of morphology (*n* = 250) **(F)**. L-Methionine alleviates cell growth **(C)** but the combination of four amino acids L-methionine, L-lysine, L-aspartate, and L-threonine resulted in cell chaining **(D)**. Addition of L-ornithine to the minimal medium also induced cell chaining **(E)**. Scale bar, 5 μm.

We further investigated if amino acids were able to delay cell separation in *D. indicus*. *D. indicus* cells propagated in M2G medium containing a combination of all 20 amino acids (Supplementary Table S1), exhibited multi-cell chains (Figure 7B). About 25% of cell population grew as chains in M2G medium supplemented with 0.1% amino acids mixture (Figure 7F). Increasing the concentration of all 20 amino acids above 0.1% did not augment cell chaining (data not shown). M2G is a minimal growth medium, containing ammonium chloride as a nitrogen source. We also grew *D. indicus* cells in M2G medium supplemented with increasing amount of ammonium chloride and the cells retained their rod shape (Supplementary Figure S2). Taken together our data reinforce the notion that concentration of amino acids in the growth medium influences the morphology of *D. indicus* cells.

To further confirm whether the cell chaining could be recapitulated by addition of any single amino acid in minimal medium, we grew *D. indicus* cells in M2G medium with exogenous amino acids. None of the 20 amino acids when supplemented individually in M2G affected cell morphology. As no single amino acid was able to recapitulate the phenotype, it is probable that a combination of more than one amino acid is required to alter cell morphology of *D. indicus*. To narrow down which combination of amino acids to test we selected the combinations listed in the Manual for Advanced Molecular Genetics (Davis et al., 1980) (Supplementary Table S1). Out of all the 11 combinations tested, only one combination consisting of L-methionine (0.9%), L-lysine (1.1%), L-aspartate (1.0%), and l-threonine (0.71%) showed cell deformities (Supplementary Tables S1, S2). M2G medium containing this combination of amino acids had increased cell width and length and some cells were also growing as chains (Figure 7D and Table 1). To assess the role of each of the four amino acids, we tested various combinations of these amino acids. M2G medium when supplemented with methionine increased the growth rate but did not alter cell morphology (Figure 7C and Table 1). Cells grown in a combination of both methionine and lysine displayed multi-cell chains, while a combination of methionine with either aspartate or threonine did not alter cell morphology. *D. indicus* grown in M2G medium supplemented with a combination of 18 amino acids devoid of only lysine and methionine did not exhibit multi-cell chains. It should be noted that cells grown in the presence of both methionine and lysine displayed less cells in chains compared to cells grown in medium supplemented with all four amino acids (Figure 7 and data not shown). Moreover, increase in average cell length was observed in presence of four amino acids (10.28 μm) when compared to control cells in M2G medium (2.5 μm) alone or M2G medium supplemented with 18 amino acids mixture lacking methionine and lysine (3.33 μm). Taken together our data suggest that the concentration of methionine and lysine in the growth medium plays a major role in inducing morphological alterations in *D. indicus* (Table 1).

**Table 1:** Cell dimensions of *D. indicus* in various amino acid concentrations grown in M2G medium.

Amino acid combinations in M2G medium	Cell width (μm) (mean ± SD)	Cell length (μm) (mean (SD)
No amino acid	1.03 (0.10	2.5 ± 0.42
18 amino acids (-Lys, -Met)	1.09 ± 0.07	3.33 ± 0.77
20 amino acids	1.30 ± 0.10	7.83 ± 2.75
Combination 4 (Lys, Thr, Asp, Met)	1.51 ± 0.20	10.28 ± 4.78
L-Orn	1.28 ± 0.10	11.60 ± 2.91

*Approximately 50 cells were analyzed for each growth media conditions by ImageJ software.*

Ornithine is an intermediate of arginine biosynthesis. The Deinococcus family is predicted to have the AAA pathway for lysine biosynthesis, and enzymes involved in lysine biosynthesis are also involved in arginine biosynthesis (Miyazaki et al., 2002; Lombo et al., 2004). Thus, it is probable that the concentration of lysine in the growth medium could be regulating the amount of ornithine in the cell. We thus hypothesized that addition of exogenous ornithine alone in minimal medium would cause morphological alterations. Consistent with this hypothesis, *D. indicus* cells grown in M2G medium supplemented with 0.005% ornithine displayed multi-cell chains (Figure 7E). Moreover, average cell length of *D. indicus* grown in M2G medium supplemented with L-ornithine was approximately four times more than control cells grown in M2G medium (Table 1).

### Growth Medium-Dependent Alterations in Cell Wall Composition of *D. indicus*

**FIGURE 8:**
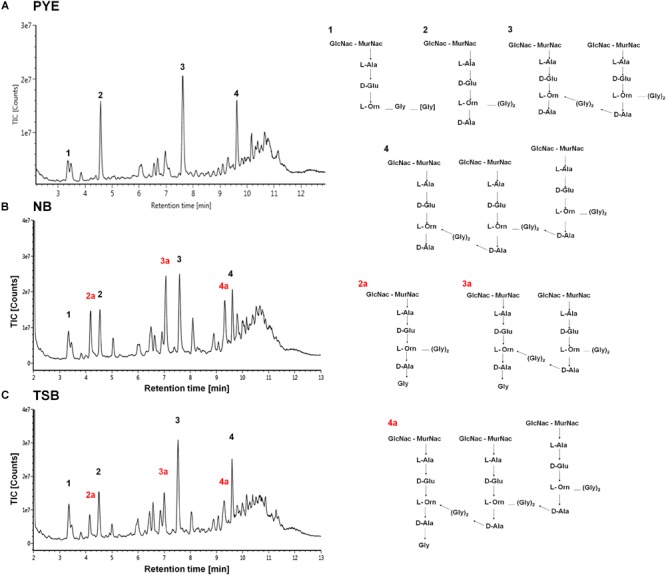
Cell wall analysis of *D. indicus* grown in different growth media. UPLC profiles of muropeptides obtained from *D. indicus* grown in PYE **(A)**, NB **(B)**, and TSB **(C)**. The chromatograms represent monomer (1 and 2), dimer (3), and trimer (4) peaks and their corresponding chemical structures are shown in right panel. Peaks 2a, 3a, and 4a correspond to penta(Gly) containing muropeptides (labeled in red).

Studies have shown that minor alterations in the composition and cross-linking of the peptidoglycan drastically affect cell shape (Nelson et al., 2002; Meberg et al., 2004; Sycuro et al., 2010; Sycuro et al., 2012; Peters et al., 2016). It is probable that growth of *D. indicus* in various media could be causing alteration in PG composition. To investigate this further a comparative analysis of PG from *D. indicus* cultures grown in either PYE, TSB, or NB was performed. As expected, we identified the presence of L-Orn muropeptides, a characteristic of Deinococcaceae family. Both L-Orn monomers and L-Orn crosslinked muropeptides were present in the samples obtained from PYE-grown cultures (Figure 8 and Supplementary Table S3). Similar to *D. radiodurans* (Quintela et al., 1999), muropeptides crosslinked by (Gly)_2_ bridges were also observed in *D. indicus* murein (Figure 8 and Supplementary Table S3). However, muropeptides from PYE grown *D. indicus* were abundant in L-Orn–D-Ala terminated muropeptides (Figure 8 and Supplementary Figure S3), which are relatively low in *D. radiodurans* (Quintela et al., 1999). PG from *D. indicus* cultures grown in nutrient-rich growth media (TSB and NB) exhibited muropeptides with extra terminal glycine [penta(Gly)] in addition to L-Orn–D-Ala terminated muropeptides (Figure 8, Supplementary Figure S3, and Supplementary Table S3). The presence of penta(Gly) peptides represented the major difference between the muropeptides profiles of *D. indicus* PG obtained from PYE, TSB, and NB cultures.

Other discernible differences were also observed upon comparison of the monomers, dimers, and trimers in PG components from various growth media. An abundance of monomers was observed in PYE medium, while an increase in trimers and dimers was seen in TSB and NB medium (Table 2). We observed an increase in dimers in NB (56%) and TSB (∼60%) media over PG from PYE (∼49%) grown cells, indicating a shift toward more cross-linked PG (Figure 8 and Table 2). An increase in the overall cross-linkage of PG was observed in nutrient-rich growth media TSB and NB (65.7 and 68.2%, respectively), whereas PYE medium displayed 57% cross-linked PG (Table 2). Taken together, our data suggest that growth medium changes lead to PG alteration in *D. indicus*.

**Table 2:** The table represents a summary of the PG composition of *D. indicus* grown in the indicated media.

	PYE	NB	TSB
Monomers	46.7	39.1	35.7
Dimers	49.2	56.7	60.3
Trimers	3.96	4.5	4
Crosslinks	57.1	65.7	68.2

*The relative molar abundance of monomers, dimers, and trimers and the total degree of crosslinking are represented in percentage. Data represented in an average of two biological replicates.*

## Discussion

In this paper, we report that low concentration of CAA in the growth medium induces changes in the cell morphology of *D. indicus*. *D. indicus* cells which are rod shaped, form multi-cell chains when grown in complex media such as LB, TSB, and NB or minimal media containing 0.1% CAA. While we are reporting growth medium-dependent morphological alteration in *D. indicus* for the first time, this phenomena is not new to the Deinococcaceae family. Chou and Tan (1991) reported salt-induced morphological changes in *D. radiodurans* cells. In *D. radiodurans* increased NaCl concentration decreased the rate of cell separation (Chou and Tan, 1991). Nutrient concentration-induced morphotypes were also observed in *D. radiodurans*, and diluted TGY medium led to growth as monomeric and dimeric units (Joshi and Toleti, 2009). *D. mumbaiensis* (now known as *D. ficus*) also displays variable morphology in different nutrient media (Shashidhar and Bandekar, 2006). *Azotobacter vinelandii* exhibits morphological variability when grown in media containing peptone. In Azotobacter, the formation of branched, giant filamentous cells was due to amino acids, particularly glycine in the medium (Vela and Rosenthal, 1972). Similarly, our data show that in *D. indicus* too, presence of CAA in minimal growth medium delayed cell separation resulting in the formation of multi-cell chains. Surprisingly, changes in sodium chloride concentration in the growth medium did not play a role in shape alteration in *D. indicus* DR1 (Supplementary Figure S1). We also found that the morphological alterations in *D. indicus* was reversible, as multi-cell chains when transferred back into nutrient-poor media reverted back to rod-shape and no longer exhibited delayed cell separation (Figure 5). Addition of all 20 amino acids to minimal media caused rod-shaped cells to grow as multi-cell chains (Figure 7). As increasing the concentration of ammonium chloride in M2G medium did not alter cell morphology, it can be concluded that cells are probably responding to increase in amino acids rather than an increase in nitrogen source (Supplementary Figure S2). Our data show that minimal medium, when supplemented with methionine, lysine, aspartate, and threonine, alters cell morphology of *D. indicus*. In these four amino acids, it was found that methionine and lysine play a more predominant role compared to aspartate and threonine (Figure 7). *Deinococcus* are phylogenetically closely related to extreme thermophiles of the genus *Thermus*. Also, both genera *Thermus* and *Deinococcus* have complex cell wall consisting of ornithine-Gly-peptidoglycan (Embley et al., 1987; Oyaizu et al., 1987; Quintela et al., 1999). Cell wall analysis of *D. indicus* revealed the presence of ornithine in the cell wall. Ornithine is generated as an intermediate in arginine biosynthesis and in Deinococcus family enzymes involved in AAA pathway for lysine biosynthesis are also involved in arginine biosynthesis (Miyazaki et al., 2002; Lombo et al., 2004). It could be predicted that concentration of lysine in the growth medium could be regulating the amount of ornithine in the cell. This notion was supported by our data, as the addition of exogenous ornithine as low as 0.005% in the growth medium was able to recapitulate the multi-cell chain phenotype.

Morphological changes in *D. indicus* may be a result of minor changes in the cell wall mediated by the concentration of amino acids in the growth medium. Similar to *D. radiodurans*, the cell wall of *D. indicus* was found to be abundant in ornithine containing muropeptides (Quintela et al., 1999). However, muropeptides from both TSB and NB showed presence of glycine terminated muropeptides, which were absent in muropeptides extracted from PYE growth medium (Figure 8). The most significant change was observed in PG components obtained from TSB medium, where the cross-linking was increased compared to PYE medium (68% vs. 57%). Studies have shown that minor alterations in the composition and cross-linking of the peptidoglycan drastically affect cell shape (Nelson et al., 2002; Meberg et al., 2004; Priyadarshini et al., 2007; Sycuro et al., 2010, 2012). Also, cell curvature might be generated by alterations in PG crosslink number or length (Huang et al., 2008). Changes in PG cross-linking lead to alteration in curvature and helicity of *Helicobacter pylori* (Sycuro et al., 2010). Alteration in the ratio of tetra–tri and tri–tri cross-links also lead to changes in surface glycopeptidolipids, colony morphology, and biofilm formation in *Mycobacterium smegmatis* (Pandey et al., 2018). Growth medium-dependent glycine incorporation in PG is also observed in *Caulobacter crescentus* (Takacs et al., 2013). However, it should be noted that in *C. crescentus* increase in pentaGly muropeptides did not alter the overall PG cross-linking (Takacs et al., 2013). Thus, *in D. indicus*, the increase in overall PG cross-linking in both TSB and NB medium could be contributing toward altered cell morphology. The increase in PG cross-linkage could be affecting the cell separation of daughter cells after division leading to formation of chains in nutrient-rich growth media.

The question still remains as to why some bacteria such as *D. radiodurans* and *D. indicus* exhibit growth media-induced morphological changes. It is proposed that bacterial size is limited by the competition for available nutrients (Young, 2006). Thus, a bacterium can adapt to nutritional stress by altering its morphological form in a particular environment. *D. indicus* delays cell separation in the presence of CAA, indicating that availability of nutrients in the environment is sufficient to induce morphological variability. By keeping the newly formed daughter cells attached in chains, *D. indicus* effectively increases the number of cells in the particular environment which probably help in establishing biofilms in natural environments. While there are reports of altered morphology in some bacteria (Chou and Tan, 1991; Rice et al., 2005; Shashidhar and Bandekar, 2006; Joshi and Toleti, 2009), spatiotemporal control of cell division and cytokinesis is still not very well understood in Deinococcaceae family. *D. indicus* can serve as a good model system to study cell division and physiology of extremophiles.

## Data Availability

All datasets generated for this study are included in the manuscript and/or the Supplementary Files.

## Author Contributions

DC and RP designed the study and performed the experiments. PS and RY performed the data analysis. BR and FC performed the muropeptides analysis. All authors contributed toward the writing of the manuscript.

## Conflict of Interest Statement

The authors declare that the research was conducted in the absence of any commercial or financial relationships that could be construed as a potential conflict of interest.

## Footnotes

1 http://www.ncbi.nlm.nih.gov/

2 http://www.ebi.ac.uk/Tools/msa/clustalo/
