# Supplementary material for: Amino Acid-Dependent Alterations in Cell Wall and Cell Morphology of Deinococcus indicus DR1
Source: Front Microbiol. 2019 Jul 3;10:1449. doi: 10.3389/fmicb.2019.01449 (PMC6618347; doi:10.3389/fmicb.2019.01449)
Supplement: Supplementary file 3 [file Data_Sheet_1.docx]

**(Supplementary data)**

**Amino Acid-Dependent Alterations in Cell Wall and Cell Morphology of *Deinococcus indicus* DR1**

**Deepika Chauhan^1^, Pulkit Anupam Srivastava^2^, Barbara Ritzl^3^, Ragothaman Yennamalli^2^, Felipe Cava^3^, Richa Priyadarshini^1*^**

^1^ Department of Life Sciences, School of Natural Sciences, Shiv Nadar University, Gautam Buddha Nagar, Uttar Pradesh, India

^2^ Department of Biotechnology and Bioinformatics, Jaypee University of Information

Technology, Waknaghat, Himachal Pradesh, India

^3^ Laboratory for Molecular Infection Medicine Sweden, Department of Molecular Biology, Umeå Centre for Microbial Research, Umeå University, Umeå, Sweden

***** Corresponding author

**Correspondence and reprints**:

**Dr. Richa Priyadarshini**

Department of Life Sciences, School of Natural Sciences, Shiv Nadar University, Gautam Buddha Nagar, Uttar Pradesh, India.

Ph no: +91-120-3819100 Ext. 220

Email: [richa.priyadarshini@snu.edu.in](mailto:richa.priyadarshini@snu.edu.in) (RP)

**Supplementary figure legends**

**Supplementary Video S1.** Time-lapse showing morphological transition from rod-shaped cells to multi-cell chains in *D. indicus.* Overnight culture in PYE broth was re-inoculated in fresh nutrient broth after double wash with 1X PBS. After 0.2 OD_600_, time-lapse was performed for 7 h in DIC at 30˚C. Rod shape of the bacterium was changed into long chain (Time-lapse corresponding to Fig 2).

**Supplementary Video S2.** Time-lapse showing morphological transition from multi-cell chains to rod-shaped cells in *D. indicus.* Cells cultured overnight in nutrient broth was washed twice and re-inoculated in PYE medium and observed under the microscope for 6 h in DIC (Time-lapse corresponding to Fig 5). Elongated chains have been altered to rods showing regular division pattern.

**Supplementary Figure S1.** Sodium chloride does not induce shape alteration. Culture was grown overnight in PYE, LB and NB media and re-inoculated next day in amended media devoid of NaCl. PYE was tested with the addition of 0.5 % NaCl. Cells were observed in DIC after 12 h of incubation at 30˚C. **A**, **B** represents PYE medium without (control) and with 0.5 % NaCl. In LB **C**, **D** and NB media **E**, **F** shows no change in morphology with and without salt respectively. Scale bar equal to 10 µm.

**Supplementary Figure S2.** Effect of nitrogen on cell morphology. *D. indicus* was inoculated in M2G and re-inoculated in M2G containing increasing amount of ammonium chloride. **A** Control, without additional NH_4_Cl; **B** 0.2%, **C** 0.4%, **D** 0.6%, **E** 0.8% NH_4_Cl. Cell shows no morphological alteration on addition of NH_4_Cl. Imaging was performed in DIC using 100X oil immersion lens after 24 hours of incubation at 30˚C, 200 rpm. Scale bar equals 5 μm.

**Supplementary Figure S3.** UPLC-MS analysis of *D. indicus* muropeptides. UPLC-MS analysis of the muropeptides 1, 2a and 2 (**A-C**). Fragmentation pattern of the muropeptides with their corresponding mass values for each fragment and the scheme of the chemical structure. (”t” refers to terminal).

|  | **1** | **2** | **3** | **4** | **5** |
| --- | --- | --- | --- | --- | --- |
| **6** | Adenosine | Guanosine | Cysteine | Methionine | Thiamine |
| **7** | Histidine | Leucine | Isoleucine | Lysine | Valine |
| **8** | Phenylalanine | Tyrosine | Tryptophan | Threonine | Proline |
| **9** | Glutamine | Asparagine | Uracil | Aspartic acid | Arginine |
| **10** | Thymine | Serine | Glutamic acid | DAP | Glycine |
|  |  |  |  |  |  |
| **11** | Pyridoxine, nicotinic acid, biotin, pentothenate, alanine |  |  |  |  |

**Table S1.** List of combinations used to determine the effect of specific amino acids. The compositions of media 1-5 are listed vertically in the table. The compositions of media 6-10 are listed horizontally. Medium 11 is an assortment of compounds not included in the others; contents listed horizontally at the bottom of the table.

**Table S2.** Amino acid concentrations used for media composition. The final concentration (200 times dilution) results in appropriate concentration of the amino acid required in growth media.

**Table S3.** Molecular mass analysis of *D. indicus* peptidoglycan muropeptides. To confirm the identity of muropeptides, the mass of individual peaks was compared with their calculated mass [Da]

**Table S4.** Generation time of *D. indicus* in various growth media.

**Table S1**

| Amino acid | Stock conc. (mg/ml)* | Final conc. (mg/ml) |
| --- | --- | --- |
| Alanine | 8.4 | 0.042 |
| Arginine | 25.3 | 0.127 |
| Asparagine | 8.4 | 0.042 |
| Aspartate | 10.0 | 0.05 |
| Cysteine | 7.3 | 0.037 |
| Glutamate | 10.0 | 0.05 |
| Glutamine | 146.0 | 0.73 |
| Glycine | 2.0 | 0.01 |
| Histidine | 3.1 | 0.015 |
| Isoleucine | 7.9 | 0.039 |
| Leucine | 7.9 | 0.039 |
| Lysine | 11.0 | 0.055 |
| Methionine | 9.0 | 0.045 |
| Phenylalanine | 9.9 | 0.049 |
| Proline | 46.0 | 0.23 |
| Serine | 84.0 | 0.42 |
| Threonine | 7.1 | 0.035 |
| Tryptophan | 4.1 | 0.020 |
| Tyrosine | 3.6 | 0.018 |
| Valine | 7.0 | 0.035 |

***1mg/ml = 0.1%**

**Table S2**

|  | [M+H]+ | |  |
| --- | --- | --- | --- |
| Peak | **Calculated Mass [Da]** | **Measured Mass [Da]** | **Mass difference [Da]** |
| 1 | 927.4391 | 927.4120 | 0.027 |
| 2a | 1055.4738 | 1055.4570 | 0.016 |
| 2 | 998.4524 | 998.4464 | 0.006 |
| 3a | 2034.9084 | 2034.9034 | 0.005 |
| 3 | 1977.8870 | 1977.8613 | 0.025 |
| 4a | 3014.3430 | 3014.3353 | 0.007 |
| 4 | 2957.3215 | 2957.3155 | 0.006 |

**Table S3**

| Medium | Generation time (min)*  (Mean + SD) |
| --- | --- |
| PYE | 120 ± 4 |
| LB | 150 ± 4 |
| NB | 90 ± 1 |
| TSB | 118 ± 6 |

**Table S4**
