# Supplementary figures and images for: Amino Acid-Dependent Alterations in Cell Wall and Cell Morphology of Deinococcus indicus DR1
Source: Front Microbiol. 2019 Jul 3;10:1449. doi: 10.3389/fmicb.2019.01449 (PMC6618347; doi:10.3389/fmicb.2019.01449)

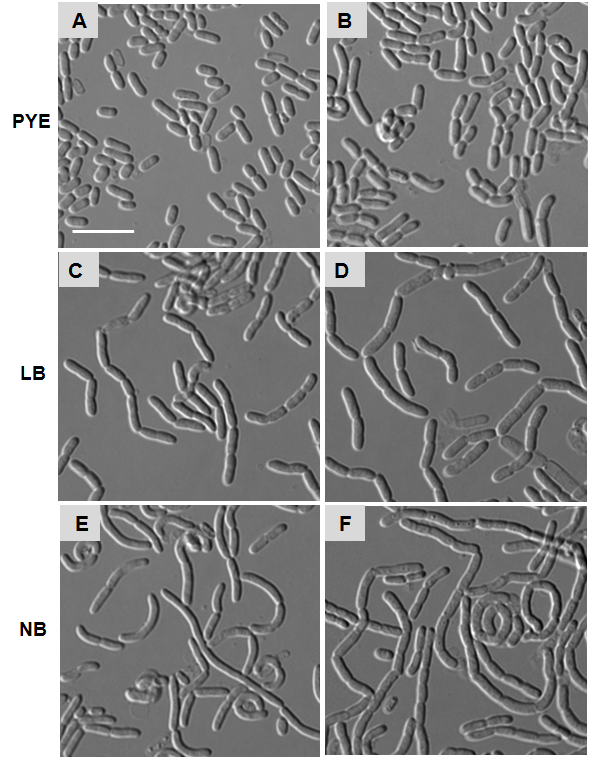

Supplement: Supplementary file 4 [file Image_1.TIF]

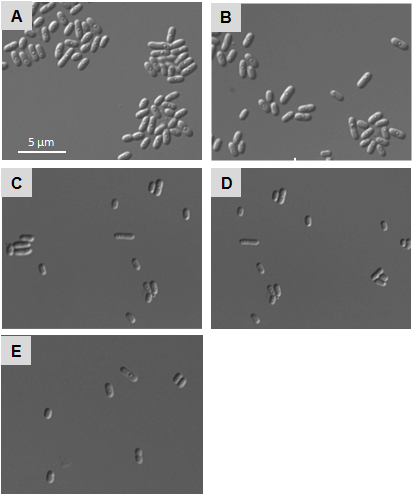

Supplement: Supplementary file 5 [file Image_2.TIF]

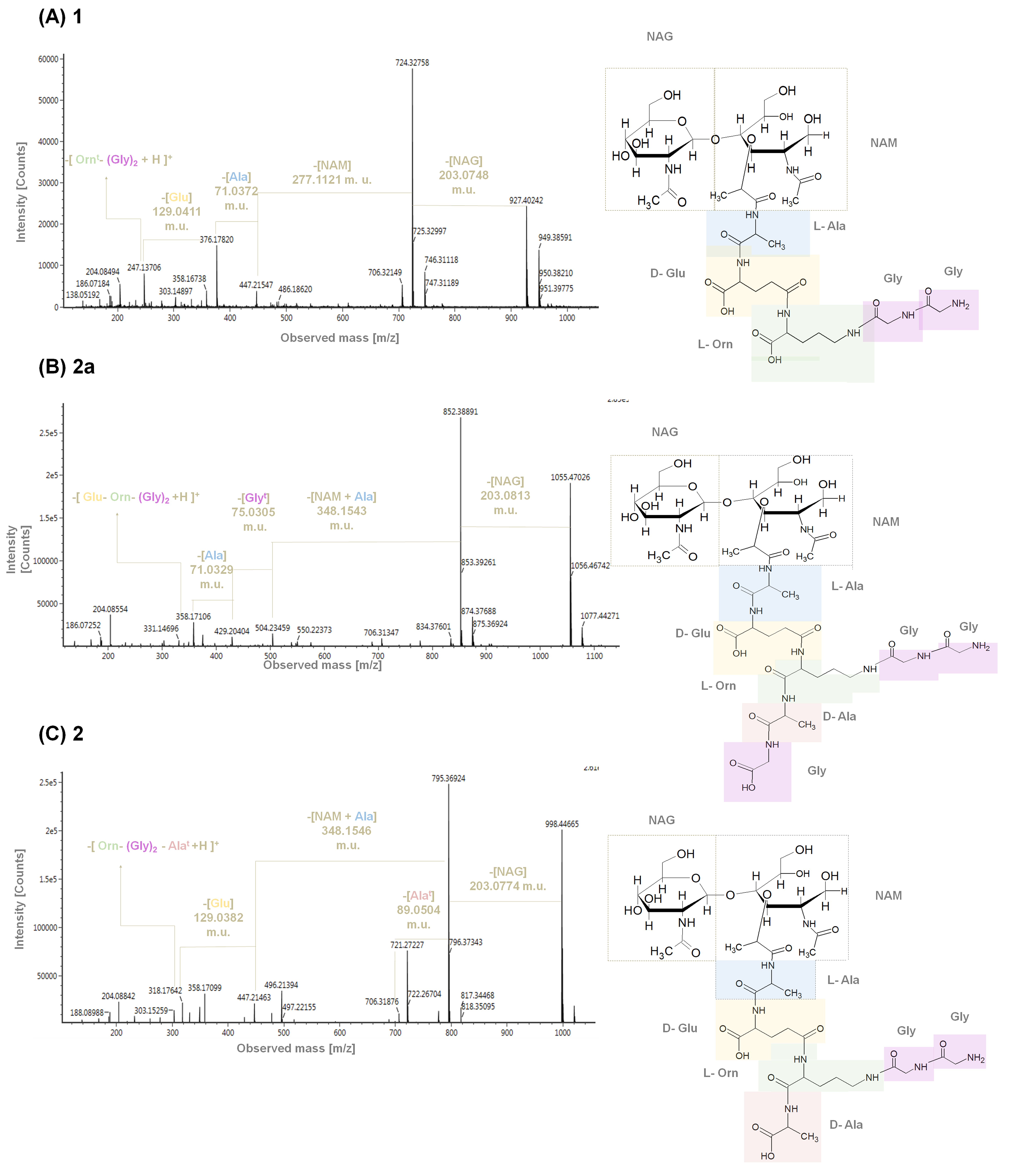

Supplement: Supplementary file 6 [file Image_3.TIF]
